# High-throughput evaluation of epilepsy-associated *KCNQ2* variants reveals functional and pharmacological heterogeneity

**DOI:** 10.1172/jci.insight.156314

**Published:** 2022-03-08

**Authors:** Carlos G. Vanoye, Reshma R. Desai, Zhigang Ji, Sneha Adusumilli, Nirvani Jairam, Nora Ghabra, Nishtha Joshi, Eryn Fitch, Katherine L. Helbig, Dianalee McKnight, Amanda S. Lindy, Fanggeng Zou, Ingo Helbig, Edward C. Cooper, Alfred L. George

**Affiliations:** 1 Department of Pharmacology, Northwestern University Feinberg School of Medicine, Chicago, Illinois, USA.; 2 Departments of Neurology, Neuroscience, Molecular and Human Genetics, Baylor College of Medicine, Houston, Texas, USA.; 3 The Epilepsy NeuroGenetics Initiative (ENGIN), and; 4 Division of Neurology, Children’s Hospital of Philadelphia, Philadelphia, Pennsylvania, USA.; 5 Invitae, Inc., San Francisco, California, USA.; 6 GeneDx, Inc., Gaithersburg, Maryland, USA.; 7 Department of Neurology, University of Pennsylvania, Perelman School of Medicine, Philadelphia, Pennsylvania, USA.; 8 Center for Pharmacogenomics, Northwestern University Feinberg School of Medicine, Chicago, Illinois, USA.

**Keywords:** Genetics, Neuroscience, Epilepsy, Pharmacology, Potassium channels

## Abstract

Hundreds of genetic variants in *KCNQ2* encoding the voltage-gated potassium channel K_V_7.2 are associated with early onset epilepsy and/or developmental disability, but the functional consequences of most variants are unknown. Absent functional annotation for *KCNQ2* variants hinders identification of individuals who may benefit from emerging precision therapies. We employed automated patch clamp recordings to assess at, to our knowledge, an unprecedented scale the functional and pharmacological properties of 79 missense and 2 inframe deletion *KCNQ2* variants. Among the variants we studied were 18 known pathogenic variants, 24 mostly rare population variants, and 39 disease-associated variants with unclear functional effects. We analyzed electrophysiological data recorded from 9,480 cells. The functional properties of 18 known pathogenic variants largely matched previously published results and validated automated patch clamp for this purpose. Unlike rare population variants, most disease-associated *KCNQ2* variants exhibited prominent loss-of-function with dominant-negative effects, providing strong evidence in support of pathogenicity. All variants responded to retigabine, although there were substantial differences in maximal responses. Our study demonstrated that dominant-negative loss-of-function is a common mechanism associated with missense *KCNQ2* variants. Importantly, we observed genotype-dependent differences in the response of *KCNQ2* variants to retigabine, a proposed precision therapy for *KCNQ2* developmental and epileptic encephalopathy.

## Graphical abstract

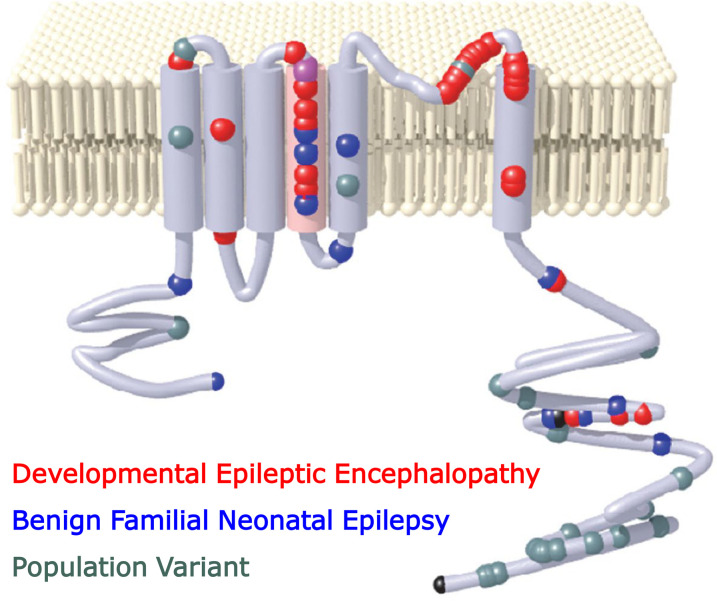

## Introduction

Pathogenic variants in voltage-gated sodium and potassium channel genes account for a large fraction of individuals diagnosed with early life epilepsy and related developmental and epileptic encephalopathies (DEEs) (1–4). Recent investigations of disease-associated variants have yielded important discoveries about the molecular mechanisms responsible for early life epilepsy and inspired a framework for individualized treatment known as precision medicine (5, 6). However, increased use of genetic testing has resulted in the ongoing discovery of a greater number of candidate pathogenic variants than can be accommodated by standard investigative approaches.

Pathogenic variants in *KCNQ2* are a frequent cause of early-onset monogenic epilepsy and neurodevelopmental disorders (7, 8). *KCNQ2* encodes a voltage-gated potassium channel subunit (KCNQ2 or K_V_7.2) that forms tetrameric channels by itself or coassembles with the related KCNQ3/K_V_7.3 subunit to generate an important, slowly-activating, noninactivating muscarinic acetylcholine receptor modulated current (M-current) in central and peripheral neurons (9–12). *KCNQ2*-related disorders are highly penetrant but exhibit phenotypic and allelic heterogeneity that makes laboratory and clinical variant interpretation challenging. Benign familial neonatal epilepsy (BFNE) is dominantly transmitted and is associated with *KCNQ2* stop-gain variants in about 70% of pedigrees (13–15). Notably, *KCNQ2* and certain *KCNQ3* variants cause BFNE that is phenotypically indistinguishable. By contrast, other *KCNQ2* variants, usually missense, or, more rarely, small inframe deletions arising de novo, were identified in persons with a spectrum of more disabling phenotypes, including neonatal onset seizures with subsequent moderate to severe global disability and later-onset disability with and without seizures (16–20). Elucidation of genotype-phenotype relationships along the *KCNQ2* DEE spectrum has relied on identification and multidisciplinary study of a handful of recurrent *KCNQ2* variants. These studies have revealed correlations between phenotypes and distinctive functional profiles, ranging from strong loss-of-function (21, 22) to strong gain-of-function (16–18). Although these foundational studies provided a framework for *KCNQ2* variant genotype-phenotype correlations, several hundred disease-associated *KCNQ2* variants have unknown functional consequences. The lack of functional evidence regarding these variants limits assignment of pathogenicity, leaves individual families’ diagnostic journey in limbo and may be a hurdle to enrollment into precision medicine trials.

In vitro voltage-clamp studies of heterologously expressed recombinant human K_V_7.2 channels have been informative regarding the functional consequences of disease-associated variants (14, 21–28). Although valuable, these studies have been confounded by varied experimental strategies and expression systems employed by multiple independent laboratories. This experimental heterogeneity makes direct comparison of findings for variants studied in different laboratories and by different methods challenging. Furthermore, targeted therapy of *KCNQ2*-related epilepsy with an approved small molecule M-current activator (retigabine/ezogabine) has been demonstrated (13, 29, 30), but whether all disease-associated variants will respond equally to this drug has not been determined.

In this study, we developed a pipeline for profiling key functional consequences of *KCNQ2* variants with a rigorously validated strategy using automated patch clamp recordings. This approach enabled us to compare the functional properties of disease-associated variants with rare population variants and to investigate systematically the pharmacological response of individual variants to retigabine. Our findings greatly expand knowledge regarding the molecular basis of *KCNQ2*-related epilepsy including functional comparisons among variants associated with the spectrum of clinical severity and offer new information about the heterogeneity of retigabine responsiveness that will contribute to its emerging use for precision therapy of this monogenic epilepsy.

## Results

### Variant curation and prioritization for functional screening.

We assembled a de-identified panel of disease-implicated *KCNQ2* variants from databases of individuals with early-onset epilepsy and/or neurodevelopmental impairment participating in research, individuals undergoing clinical genetic testing, and variants described in prior publications. Variants selected for high-throughput functional analysis included “benchmark variants” (recurrent variants with well-established phenotype associations and functional consequences previously determined by voltage-clamp recordings in heterologous cells), variants with established pathogenicity (recurrent de novo variants in affected individuals with characteristic *KCNQ2*-associated phenotypes) but without previous functional analysis, and rare variants with less certain pathogenicity. Rare variants present in gnomAD with minor allele frequencies higher than expected for pathogenic variants were included as a presumed nonpathogenic control group. The complete variant panel with associated phenotypes, channel domain, minor allele frequency, database, and literature references (where applicable) is provided (see Supplemental Table 1; supplemental material available online with this article; https://doi.org/10.1172/jci.insight.156314DS1) and location with the KCNQ2 channel protein for each variant are illustrated in Supplemental Figure 1).

### Validation of automated patch clamp for assessing KCNQ2 variants.

**Figure 1:**
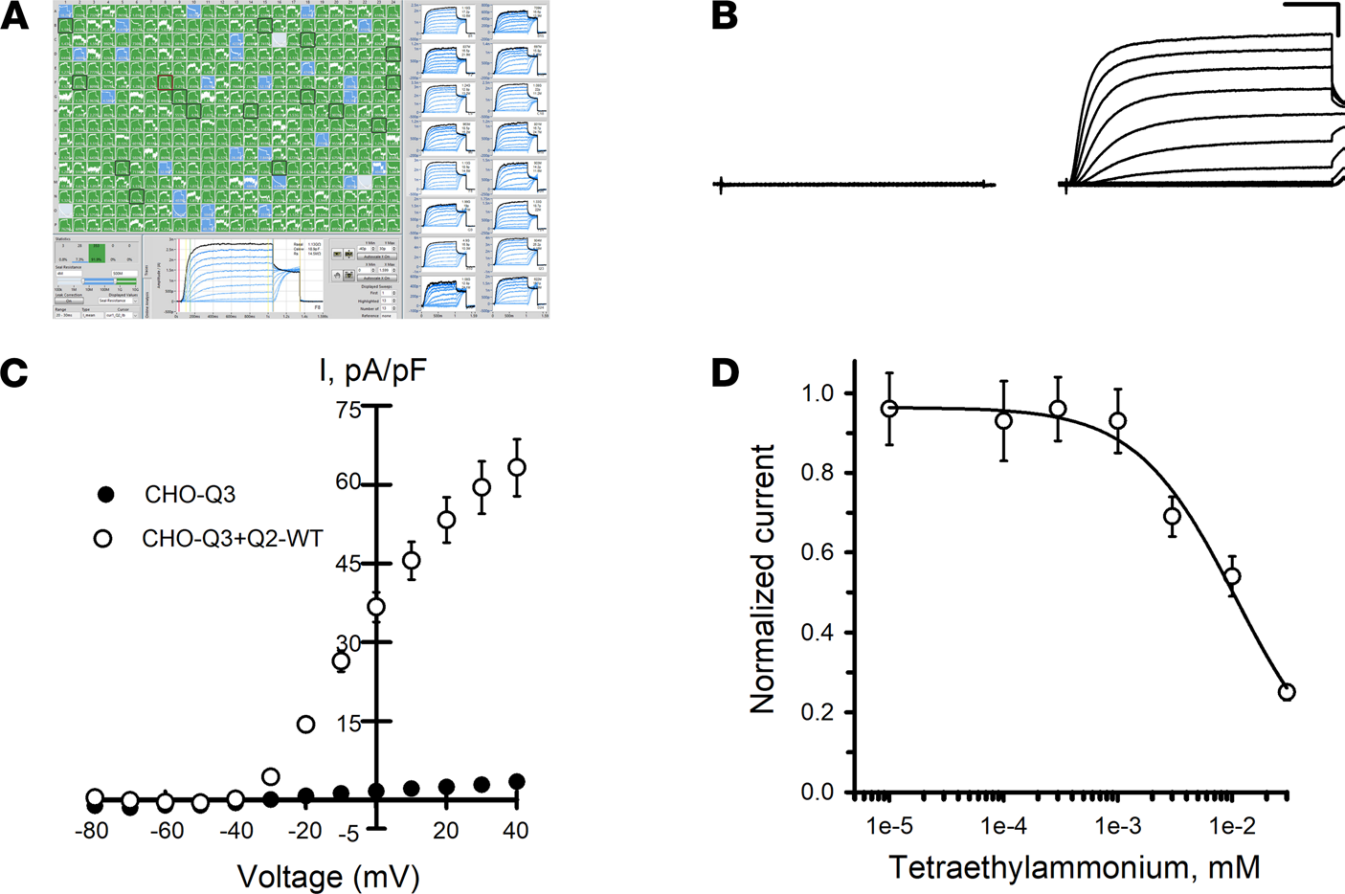
Functional analysis of KCNQ2/KCNQ3 channels by automated patch clamp. (**A**) Screen display from automated patch clamp experiment illustrating whole-cell current recordings from CHO-Q3 cells transiently expressing KCNQ2 variants (6 variants, 4 columns per variant). (**B**) Averaged XE991-sensitive whole-cell currents recorded from nontransfected CHO-Q3 cells and CHO-Q3 cells electroporated with WT KCNQ2. (**C**) Average current-voltage relationships measured from nontransfected (filled circles, *n* = 94) or KCNQ2-WT–transfected (open circles, *n* = 124) CHO-Q3 cells. Current recorded from each cell was normalized to cell capacitance as a surrogate for cell size to calculate current density (I, pA/pF). (**D**) Concentration-response relationship for TEA block of whole-cell currents recorded from CHO-Q3 electroporated with KCNQ2-WT (IC_50_ = 10.7 ± 4.2 mM, *n* = 51–82 for each concentration). Data shown in **C** and **D** are mean ± SEM (error bars are smaller than some data symbols). Scale bars: 200 ms (horizontal); 20 pA/pF (vertical).

**Figure 2:**
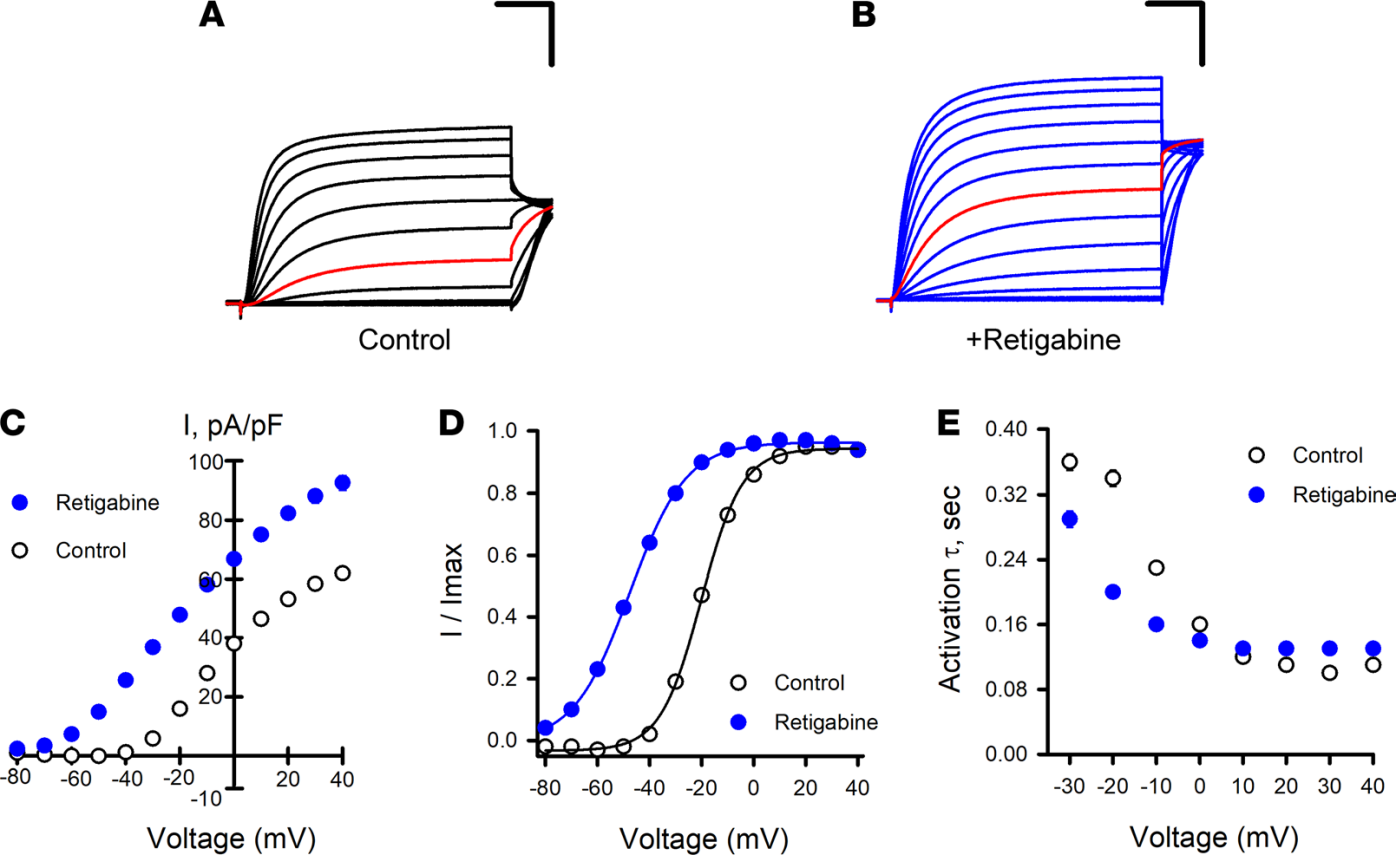
Effects of retigabine on KCNQ2/KCNQ3 channel activity. (**A**) Averaged XE991-sensitive whole-cell currents normalized by membrane capacitance recorded from CHO-Q3 cells electroporated with KCNQ2-WT exposed to control or (**B**) exposed to 10 μM retigabine solutions. Red lines indicate currents recorded at –20 mV. (**C**) Average current-voltage relationships measured from CHO-Q3 cells electroporated with KCNQ2-WT exposed to control (open circles, *n* = 1086) or retigabine (filled blue circles, *n* = 1141) solutions. (**D**) Voltage-dependence of activation measured in control (open circles, black lines) or retigabine (filled blue circles, blue lines) solutions (control: V½ = –18.9 ± 0.2, *k* = 7.6 ± 0.1, *n* = 833; retigabine: V½ = –47.7 ± 0.3, *k* = 9.9 ± 0.1, *n* = 885). (**E**) Activation time constants measured in control (open circles, *n* =525–970) or retigabine (filled blue circles, *n* = 1036–1079) solutions. Scale bars: 200 ms (horizontal); 20 pA/pF (vertical). Data shown in **C–E** are mean ± SEM (error bars are smaller than some data symbols).

To enhance reproducibility in studies of KCNQ2/KCNQ3 (Q2/Q3) heteromeric channels, we generated a Chinese hamster ovary cell line that stably expresses WT human KCNQ3 (CHO-Q3 cells) and then used electroporation with combinations of WT and variant KCNQ2 plasmids to express KCNQ2/KCNQ3 (Q2/Q3) heterotetramers. Automated patch clamp recordings demonstrated that maximal (peak) current density measured at +40 mV from CHO-Q3 cells electroporated with WT KCNQ2 was approximately 20-fold larger than that measured from nontransfected cells (Figure 1, A–C). The lack of current obtained from cells expressing KCNQ3 alone suggested that currents from KCNQ2-electroporated CHO-Q3 cells were likely Q2/Q3 heterotetramers. Indeed, the sensitivity of the measured currents to tetraethylammonium (TEA) ion (IC_50_, 10.7 ± 4.2 mM; Figure 1D) is consistent with previous reports of TEA sensitivity for Q2/Q3 channels expressed in CHO cells, and unlike the sensitivity for either homotetrameric KCNQ2 (0.2 mM) or KCNQ3 (> 100 mM) channels (31, 32). Currents recorded from the same cells by conventional manual patch clamp exhibited similar voltage-dependent gating properties (Supplemental Figure 2). Differences in current density between manual and automated methods appeared related to differences in sampling (e.g., use of GFP expression to select higher expressing cells for manual patch clamp recording). Finally, we tested the effects of retigabine on Q2/Q3 channel activity (Figure 2). Retigabine (10 μM) induced a 1.4-fold increase in peak current density measured at +40 mV accompanied by a strong hyperpolarizing shift (V½ shifted –29.2 ± 0.3 mV) in the voltage-dependence of activation and faster activation kinetics in the –30 to –10 mV range, in agreement with previous reports (32–35).

**Figure 3:**
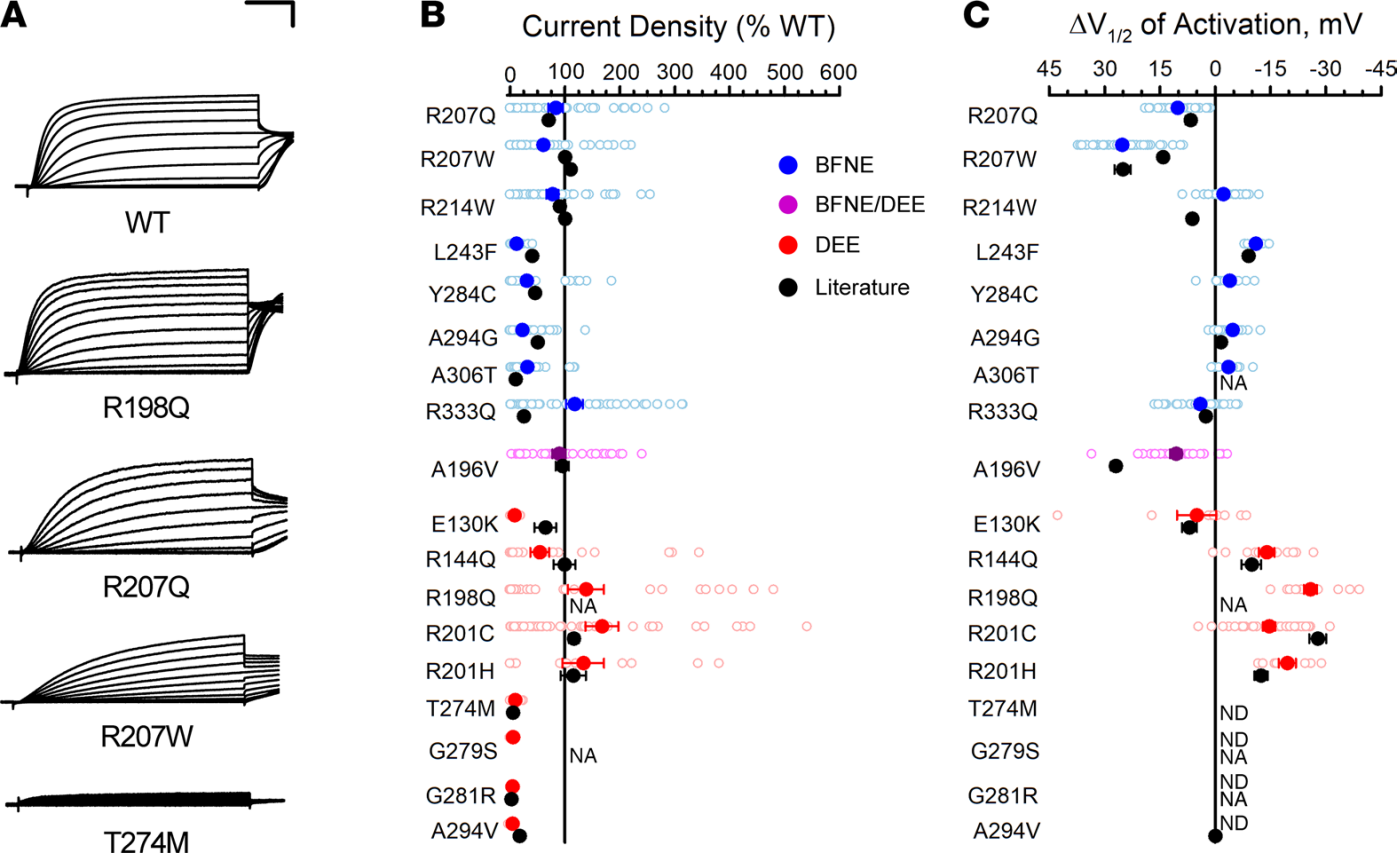
Functional properties of homozygous KCNQ2 variants determined by automated patch recording are comparable to those from previous voltage-clamp studies. (**A**) Examples of averaged XE991-sensitive whole-cell currents recorded by automated patch clamp from CHO-Q3 cells electroporated with select KCNQ2 variants. Current values were normalized to WT channel peak current that was measured in parallel. Scale bars: 200 ms (horizontal); 25% (vertical). (**B**) Average peak whole-cell currents recorded at +40 mV from cells coexpressing KCNQ3 and KCNQ2 variants displayed as percent of WT channel that was measured in parallel. (**C**) Difference in activation V½ determined for cells coexpressing KCNQ3 and KCNQ2 variants relative to WT channel (horizontal scaling was designed to show loss-of-function in the leftward direction from zero). All experimental data are presented in **B** and **C** as open circles with filled circles representing mean values. Black symbols represent mean ± SEM voltage-clamp data from literature reported variants (error bars are smaller than data symbol in some cases), while automated patch clamp results are shown as blue for BFNE, red for DEE, or purple symbols for BFNE/DEE pathogenic variants. NA, not available; ND, cannot be determined.

To demonstrate that automated patch clamp recording is a valid approach for evaluating the functional consequences of epilepsy-associated *KCNQ2* variants, we compared data we obtained for 18 variants to results from prior voltage-clamp recording in either mammalian cells or *Xenopus* oocytes (13, 16, 21, 23–25, 28, 36–41). To enable comparison of channel properties resolved by different experimental approaches, we quantified differences in peak current density and the midpoint of activation voltage dependence (V½) relative to WT channels assayed in parallel. Low current densities of KCNQ2 homomeric channels (i.e., absence of KCNQ3) in our system precluded study of this channel type. Instead, we either electroporated each variant alone in the CHO-Q3 cells, mimicking homozygosity, or electroporated a 1:1 ratio of WT and variant KCNQ2, mimicking heterozygosity. We then compared the results obtained for each variant with prior work in the homozygous and heterozygous states (complete data set presented in Supplemental Table 2). Averaged whole-cell currents normalized to peak current amplitude measured for WT channels were recorded from literature variants in the homozygous (Supplemental Figure 3) and heterozygous (Supplemental Figure 4) configurations. Results were concordant between automated and manual patch clamp recording for the majority of the 18 literature variants despite the diversity of laboratories and expression systems (Figure 3). This included variants with loss-of-function and gain-of-function effects. There were divergent results for only 2 BFNE variants — peak current density for R333Q in the homozygous state (Figure 3) and L243F in the heterozygous state (Supplemental Figure 5) — and small quantitative differences for a small number of other variants. We concluded that automated patch clamp recording was a robust and valid strategy to assess the functional properties of KCNQ2 variants.

### Functional analysis of rare population variants.

**Figure 4:**
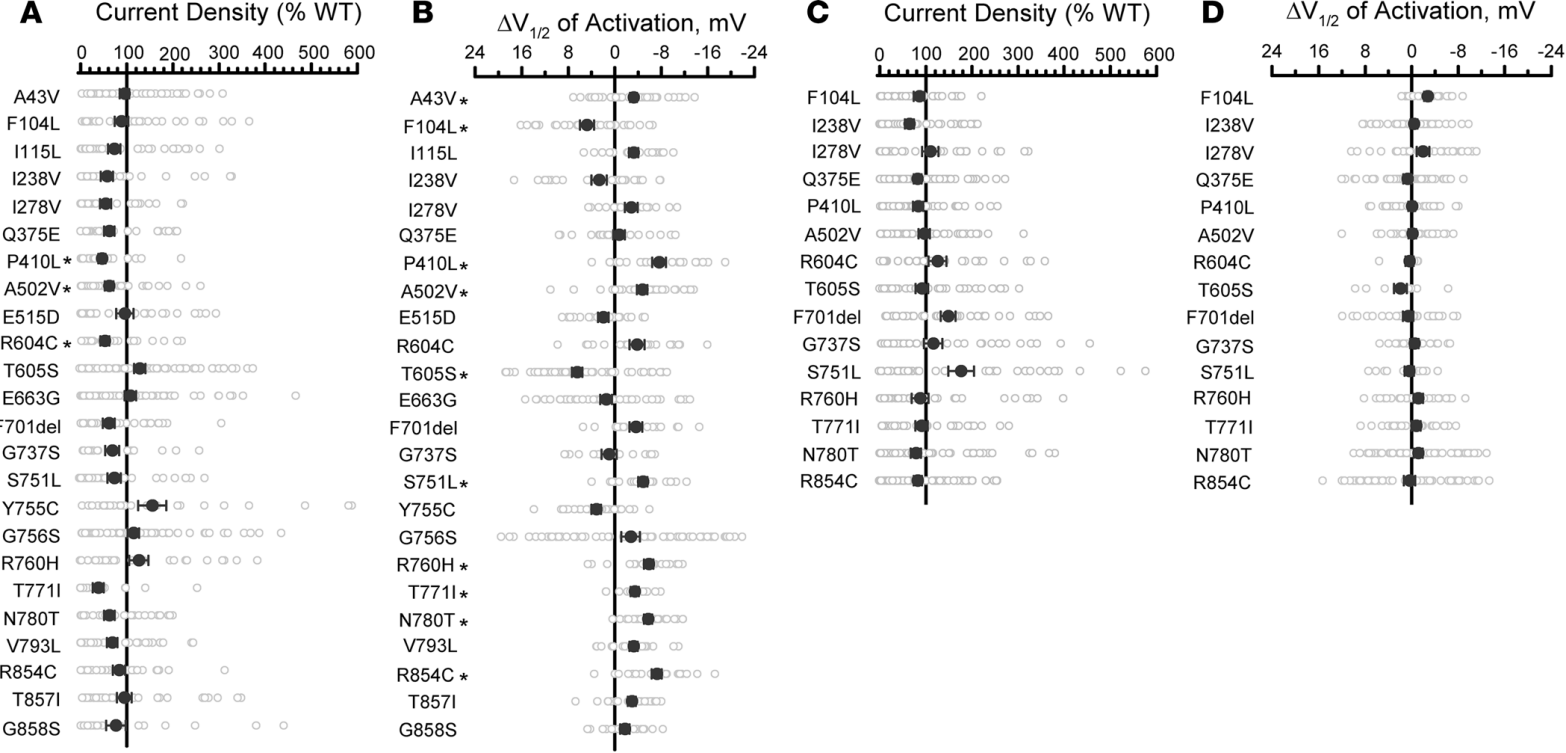
Functional properties of KCNQ2 population variants. (**A**) Average whole-cell currents recorded at +40 mV expressed as percent of the WT channel (*n* = 22–71). KCNQ2 variants were expressed in the homozygous state in CHO-Q3 cells. (**B**) Differences in activation V½ (*n* = 15–60) relative to the WT channel determined for KCNQ2 population variants. Full data sets for **A** and **B** are provided in Supplemental Table 3. (**C**) Average whole-cell currents recorded at +40 mV (*n* = 27–71) for select population variants expressed in the heterozygous state in CHO-Q3 cells. Only variants exhibiting significantly different properties from WT in the homozygous state were examined. (**D**) Differences in activation V½ (*n* = 9–59) relative to the WT channel determined for select population variants expressed in the heterozygous state. All experimental data are presented as open circles with filled circles representing mean values. Statistical significance was determined by 1-way ANOVA. *P ≤ 0.01. Full data sets for **C** and **D** are provided in Supplemental Table 4.

To assess KCNQ2 functional variation in the general population, we studied 24 population variants obtained from the gnomAD database (42) (Supplemental Table 1). Most of these variants are localized to cytosolic domains (17 C-terminus, 1 N-terminus) whereas 3 variant residues lie within transmembrane segments or an intersegment linker region (S1–S2). All except one of these variants are rare and only present heterozygously in gnomAD. The exception was the common variant N780T, which has an allele frequency of 0.50–0.66 (homozygosity frequency 0.25–0.43) among population subgroups defined in gnomAD. We first screened all the variants in the homozygous state and then studied the variants in the heterozygous state only if they exhibited significant functional differences from WT. Averaged whole-cell currents for the population variants expressed in the homozygous state are shown in Supplemental Figure 6, and differences in peak current density or voltage dependence of activation V½ compared with WT channels are summarized in Figure 4, A and B (complete data set presented in Supplemental Table 4). No variant showed large differences in gating kinetics, but 5 variants (I238V, P410L, A503V, R604C and T771I) exhibited significantly smaller peak current densities (40–60%, Figure 4A) compared with the WT channel, and 8 variants had significant (≥ 5 mV) shifts in activation V½ in either the depolarizing (loss of function: F104L and T605S) or hyperpolarizing (gain of function: P410L, A503V, S751L, R760H, R854C, and common variant N780T) direction (Figure 4B and Supplemental Table 3). However, when expressed in the more clinically relevant heterozygous state, none of the rare population variants exhibited significantly lower current density or significant shifts in activation V½ (Figure 4, C and D, and Supplemental Figure 7). However, 2 rare population variants F701del and S751L (minor allele frequencies 0.9 × 10^-5^ and 6.5 × 10^-5^, respectively) had significantly greater peak current densities when studied in the heterozygous (but not in the homozygous) state. These data provide evidence that in the general population, loss-of-function is unusual with heterozygous KCNQ2 variants.

### Functional analysis of epilepsy-associated KCNQ2 variants.

**Figure 5:**
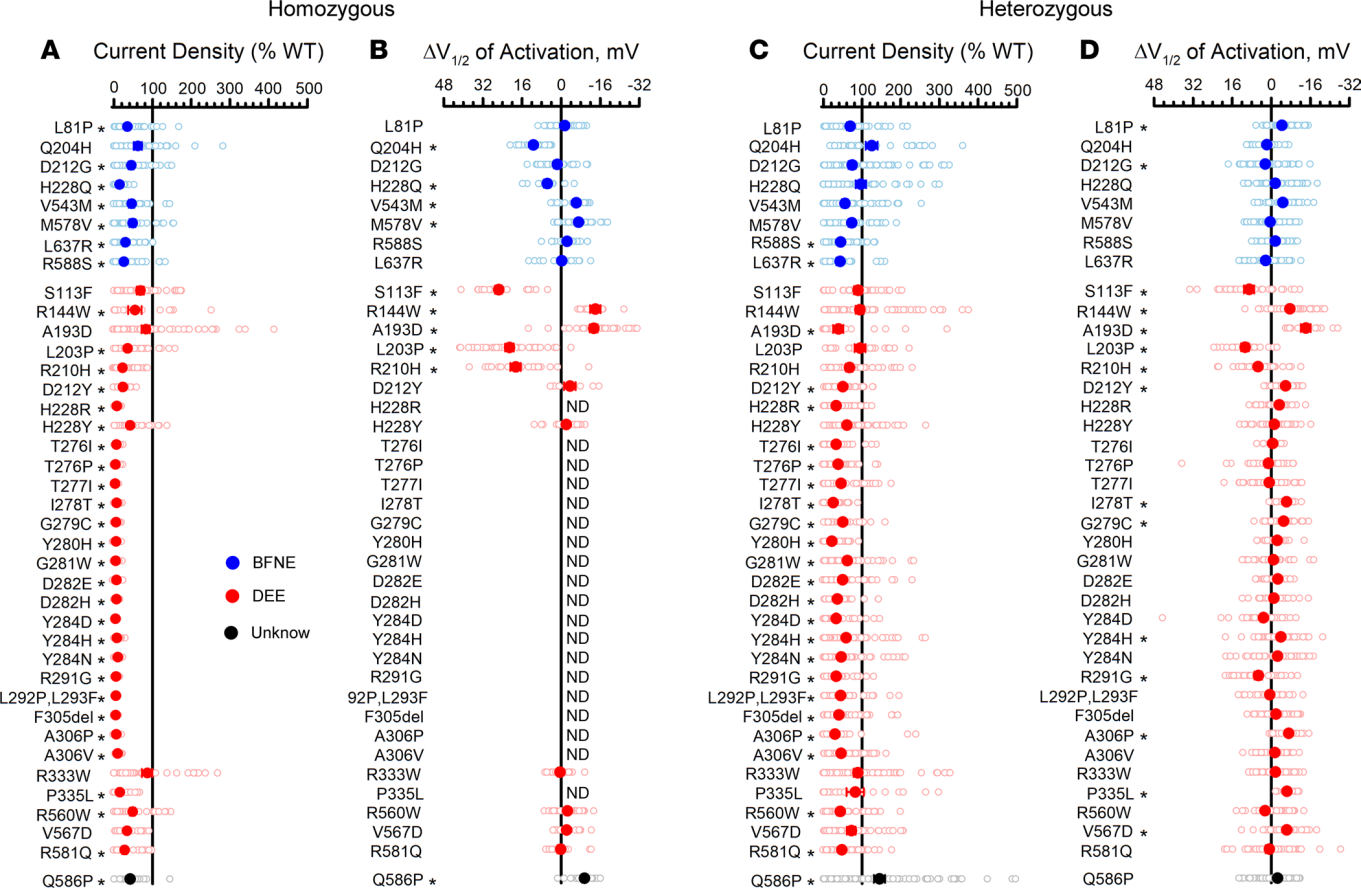
Functional properties of epilepsy-associated KCNQ2 variants. (**A**) Average whole-cell currents recorded at +40 mV from CHO-Q3 cells electroporated with epilepsy-associated KCNQ2 variants expressed in the homozygous state displayed as percent of the WT channel measured in parallel (*n* = 16–75). (**B**) Differences in activation V½ determined for disease-associated variant channels expressed in the homozygous state and quantified relative to the WT channel measured in parallel (*n* = 7–41). (**C**) Average whole-cell currents recorded at +40 mV for epilepsy-associated KCNQ2 variants expressed in the heterozygous state quantified relative to the WT channel measured in parallel (*n* = 19–84). (**D**) Differences in activation V½ determined for epilepsy-associated KCNQ2 variants expressed in the heterozygous state relative to the WT channel measured in parallel (*n* = 13–69). All experimental data are presented as open circles with larger filled circles representing mean values. Blue symbols indicate variants associated with BFNE, red symbols are variants associated with DEE, and the black symbols represent a variant with an unclear phenotype. ND, cannot be determined. Statistical significance was determined using 1-way ANOVA. *P ≤ 0.01. Full data sets are provided in Supplemental Table 3 (homozygous) and Supplemental Table 4 (heterozygous).

We determined the functional properties of 39 additional epilepsy-associated KCNQ2 variants that had not been investigated at the time of our experiments (8 BFNE, 30 DEE, 1 unknown phenotype; Supplemental Figure 1 and Supplemental Table 1). Averaged whole-cell currents recorded from CHO-Q3 cells expressing each variant in the homozygous state are presented in Supplemental Figure 8, and differences in peak current density or voltage dependence of activation V½ relative to the WT channel are summarized in Figure 5, A and B (complete data set presented in Supplemental Table 3). The majority of the variants exhibited severe loss-of-function (< 25% peak current density compared with WT channels) with profound loss-of-function (≤ 10% of WT) observed for 18 variants. The remaining variants exhibited less severe degrees of dysfunction (peak current density 25–75% of WT) or peak current density that was not significantly different from WT. None of the 39 variants exhibited current density larger than WT channels. For some variants with tail currents large enough to determine the voltage-dependence of activation, we observed significant differences from WT channels with shifts in activation V½ values in either the depolarizing (loss-of-function) or hyperpolarizing (gain-of-function) direction (Figure 5B). A depolarized activation V½ was observed for variants Q204H, H228Q, S113F, L203P, and R210H, whereas variants V543M, M578V, R144W, A193D, and Q586P exhibited hyperpolarized shifts in V½. In addition, the time course of activation was not affected by most variants with the exception of S113F and A193D, which had 3-fold and 1.5-fold slower activation, respectively (see activation time constants in Supplemental Table 3). Only the R333W variant was not significantly different from WT channels in any functional property, using a stringent threshold for significance. Importantly, there were no systematic differences in the functional properties of the tested missense variants associated with BFNE or DEE.

Because pathogenic KCNQ2 variants associated with epilepsy are heterozygous, we assessed the functional properties of each variant expressed in this context by cotransfecting CHO-Q3 cells with equal amounts of WT and variant KCNQ2 plasmids. Averaged whole-cell currents recorded in these experiments are presented in Supplemental Figure 9 and the differences in peak current density or activation V½ relative to the WT channel are summarized in Figure 5, C and D (complete data set presented in Supplemental Table 4). Dominant-negative behavior, which we defined as peak current density less than 50% of WT heterotetrameric channels (denoted as DN in Supplemental Table 4), was observed for several of the variants we studied and was more prevalent among DEE then BFNE variants. Some variants exhibited significantly smaller current density than WT channels but to a lesser degree than dominant-negative variants (V543M, H228Y, G281W, and Y284H) and this partial rescue (denoted as pR in Supplemental Table 4) is more consistent with haploinsufficiency. Most of the remaining variants showed no difference in peak current density (full rescue [fR] in Supplemental Table 4) compared with WT channels and 1 variant (Q586P) exhibited larger current density. As observed in the homozygous state, many variants exhibited significant shifts in activation V½ relative to the WT channel. Some of the variants that lacked a significantly different peak current density exhibited significant shifts in activation V½ relative to the WT channel (R144W, P335L, and V567D), while 3 variants (Q204H, H228Q, and R333W) exhibited no significant differences in peak current density or activation V½ in the heterozygous state relative to the WT channel.

### Effects of retigabine on KCNQ2 variants.

To assess the pharmacological consequences of KCNQ2 variants, we tested the acute effects of the positive allosteric modulator retigabine (10 μM) on channel function. When variants were expressed in the homozygous state, we observed a wide range of retigabine effects on maximal current density and activation V½ (Supplemental Figure 10; complete data set in Supplemental Table 5). Some variants exhibited little to no response, whereas others showed large effects equal to or greater than WT channels. Some variants located in the S4 segment and S4-5 linker (R198Q, H228Q, and H228Y) exhibited a partial response characterized by a substantial hyperpolarizing shift in activation V½ but without a WT-like increase in current density. For S4 variants R207W and R207Q, which cause a depolarized shift of activation V½ and a marked slowing of channel opening rates across voltages (Figure 3), retigabine countered both effects, resulting in a greater relative increase in current density than observed for WT channels. Variants largely unresponsive to retigabine involved residues between 280 and 306, which encompasses portions of the pore domain from the ion selectivity filter to the nearby extracellular half of the S6 segment. These data provide a map of residues with strong effects on ion conduction that cannot be overcome by retigabine when expressed in the homozygous context.

**Figure 6:**
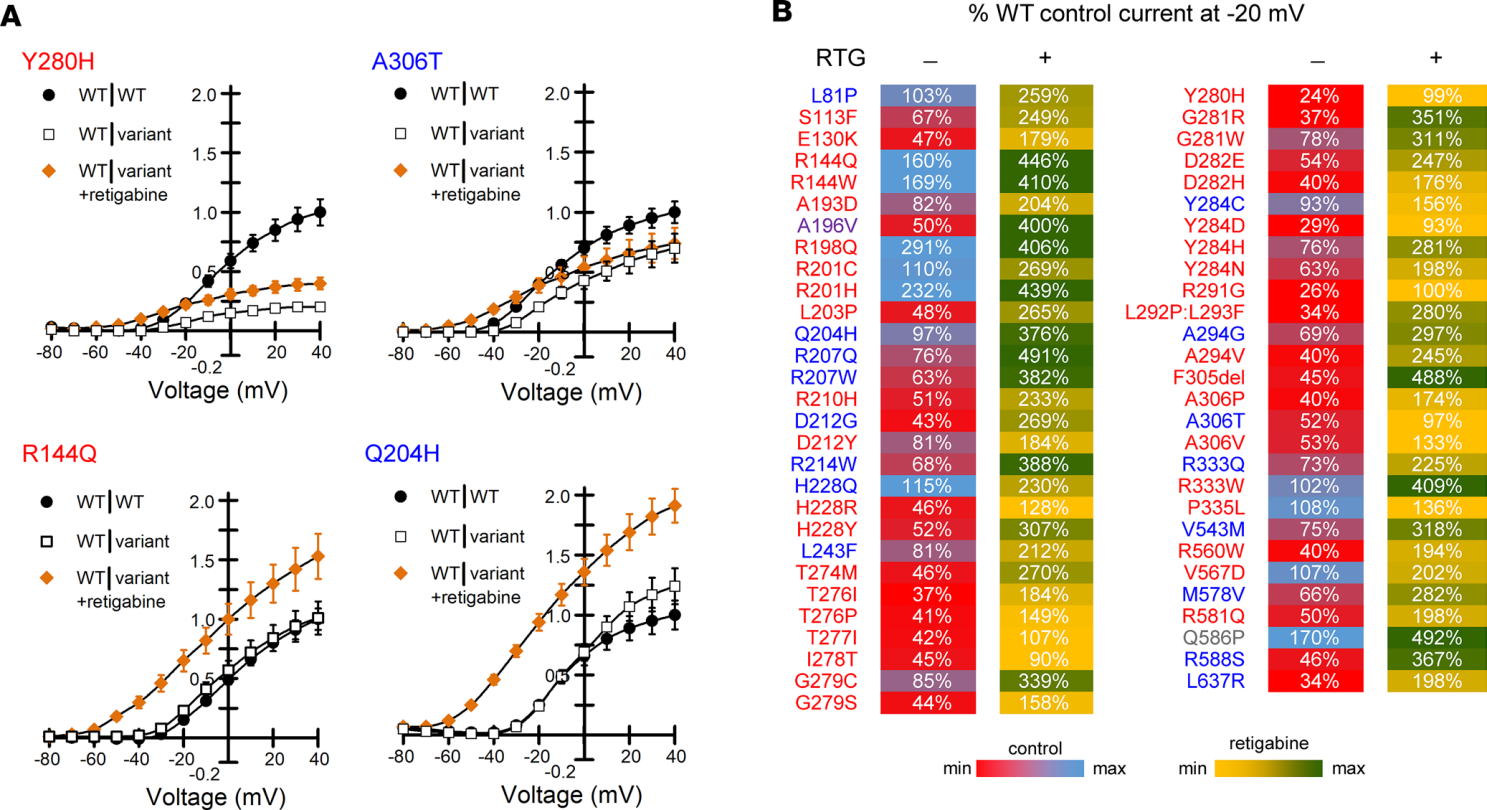
Responses of epilepsy-associated KCNQ2 variants to retigabine. (**A**) Representative current-voltage relationships comparing WT (WT|WT, filled circles) and heterozygous variant (WT|variant, open squares) channel function in the absence of retigabine with heterozygous variant function measured in the presence of 10 μM retigabine (WT|variant +retigabine, orange filled diamonds). Current amplitude was first normalized to cell capacitance, then normalized to the WT current density measured at +40 mV. Variant labels are color coded by phenotype (blue, BFNE; red, DEE; purple, BFNE/DEE; gray, unclear phenotype). Data shown are mean ± SEM (error bars are smaller than some data symbols). (**B**) Heat map summarizing retigabine response data. Control values are current density measured at –20 mV for heterozygous variants in the absence of retigabine expressed as a percentage of untreated WT channel current density (Supplemental Table 5). Retigabine values are current density measured at –20 mV for heterozygous variants in the presence of 10 μM retigabine and expressed as a percentage of untreated WT channel current density (Supplemental Table 6). Each value is colored based on the scale shown.

Because retigabine is a potential therapeutic agent for KCNQ2-associated epilepsy, we investigated its effect on epilepsy-associated variants expressed in the heterozygous state (Supplemental Table 6). We compared current density measured across a range of voltages for each heterozygous variant in the presence of retigabine with that of the WT channel recorded in the absence of drug (Supplemental Figure 11). Retigabine restored current density to at least the level of untreated WT channels for all epilepsy-associated variants at voltages more negative than the activation V½ of WT channels. However, as predicted by the results from studying homozygous channels, the effect of retigabine on current density at strongly depolarized voltages varied considerably among variants. Variants affecting residues within the ion selectivity filter (TIGYG) that exerted strong dominant-negative effects (e.g., Y280H, A306T; Figure 6A) exhibited the smallest responses to retigabine throughout the voltage range (Supplemental Figure 11 and Supplemental Table 6). Several well-established gain-of-function variants within the voltage-sensor domain (R144Q, R198Q, and R201H) and others elsewhere in the channel were hyper-responsive to retigabine (examples shown in Figure 6A, Supplemental Figure 11, and Supplemental Table 6). A summary of effects of retigabine on current density measured at –20 mV for all epilepsy-associated variants is presented as a heat map in Figure 6B illustrating that in vitro responses to retigabine exhibit considerable heterogeneity among variants.

## Discussion

Advances in understanding monogenic forms of epilepsy have motivated an aspirational approach in which treatments are designed to target specific genes or specific genetic variants (43). Before the potential of this precision medicine paradigm can be realized, a more comprehensive understanding of the pathophysiological consequences of genetic variants is needed. This requires expanding the throughput and capacity for evaluating the functional and pharmacological properties of hundreds, or even thousands, of variants using well-validated approaches, then implementing these experimental platforms for assessing genotype-specific drug responses. Additionally, data from functional studies may provide evidence of deleterious effects on the affected protein that can be useful in variant classification schemes (44).

In this study, we undertook the largest single effort to determine the functional and pharmacological properties of epilepsy-associated *KCNQ2* variants in both the homozygous and heterozygous states. The, to our knowledge, unprecedented scale of this work was made possible by using automated patch clamp recordings rather than traditional voltage clamp methods, which have limited throughput. The automated patch clamp has specific advantages over manual methods including higher throughput, unbiased cell selection, and efficient coupling to pharmacological studies. The higher throughput enables larger numbers of replicates, which strengthens statistical power and experimental rigor.

Demonstrating deleterious consequences of genetic variants assessed by well-established in vitro or in vivo methods is considered strong evidence supporting pathogenicity in the variant classification framework developed by the American College of Medical Genetics and Genomics (ACMG) (44). Inclusion of multiple control variants is critically important to ensure reliability for a functional assay (45). We validated use of automated patch clamp for KCNQ2 by performing initial experiments on 18 epilepsy-associated variants previously shown to have deleterious functional consequences, and our results were largely concordant with the prior work. We also examined 24 mostly rare population variants expected to exhibit properties similar to WT as controls. Therefore, our method satisfies these published criteria for a well-established functional assay. Interestingly, among the population variants studied, 3 exhibited modest functional differences, but the anonymity of gnomAD participants precludes our ability to make genotype-phenotype correlations.

After validating our approach, we investigated 39 epilepsy-associated variants with unknown functional consequences. These included variants with established clinical pathogenicity based on clear segregation in families or recurrence as de novo variants in unrelated individuals, as well as variants with uncertain pathogenicity. We observed that the majority of these KCNQ2 epilepsy-associated variants exhibit properties consistent with loss-of-function that can be predicted to cause impaired neuronal M-current. A small number of the epilepsy-associated variants studied here exhibited functional properties that were not dramatically different from WT channels. There are multiple potential explanations for this finding. As our sample included variants of uncertain significance on clinical grounds, some may not be disease causing and their discovery in persons with epilepsy may be incidental. Although demonstrating normal function in heterologous cells is an important observation, some variants may only have abnormal behavior in neurons. Lastly, some variants have been demonstrated to exhibit their strongest functional effects when expressed in the absence of KCNQ3 (e.g., as KCNQ2 homotetramers) (41). Unfortunately, we were unable to measure currents in cells expressing KCNQ2 only probably because of the inclusion of fluoride ions in the internal solution necessary to enhance seal formation. Fluoride ions may activate phospholipases that can degrade phosphatidylinositol 4,5-bisphosphate (PIP_2_) (46, 47), which is needed for maximal channel activity. Channel inhibition by free intracellular Mg^2+^ ions (0.57 mM in our experiments) is a less likely explanation (48).

KCNQ2 loss-of-function has many potential underlying mechanisms, including impaired trafficking to the plasma membrane, impaired subcellular localization, impaired ion conduction, and impaired activation gating (12, 21, 39, 40, 49, 50), which are not mutually exclusive. Investigating surface expression and subcellular targeting of the variants in our study may help distinguish among these possibilities.

Our study uniquely investigated the effects of retigabine on several KCNQ2 variants, and these data allowed us to recognize the variable nature of this drug response among variants. Retigabine was initially approved for adjunctive treatment of partial epilepsy in adults (32) but was withdrawn from the market because of limited use in part because of an adverse blue discoloration of skin (51). However, the drug may have potential value as targeted therapy for KCNQ2-associated epilepsy (13, 29, 30). Retigabine acts to accentuate M-current through altering the voltage-dependence of activation of KCNQ2/KCNQ3 channels. The drug binds to a hydrophobic pocket comprised of residues from both channel subunits including a tryptophan residue at position 236 in KCNQ2 and neighboring residues (e.g., Leu-299, Ser-303, Phe-305) in the 3-dimensional structure (52–55). None of the variants we studied affect residues known to be involved directly in retigabine binding.

We initially examined the effects of retigabine on KCNQ2 variants expressed in the homozygous state and observed a wide range of responses. These observations may contribute to advancing understanding of structure-pharmacology relationships for KCNQ2/KCNQ3 channels. We also conducted experiments to determine the response of epilepsy-associated variants expressed in the heterozygous state to ascertain how retigabine affects channel activity in a clinically relevant context. By comparing current density of retigabine modulated variant channels with unmodulated WT channels at a membrane potentially relevant to the physiological contribution of M-current on neuronal excitability, we observed that the function of all epilepsy-associated variants we tested could be restored to at least the level of WT channels. In some cases, current density was boosted to levels exceeding WT channels particularly for variants exhibiting gain-of-function effects in the absence of drug. However, there were large differences in the effects of retigabine on heterozygous channel current density at more depolarized potentials. The correlation between these in vitro data and the clinical response to retigabine remains to be determined. Unfortunately, this correlation is currently hindered by the paucity of published clinical drug response data, heterogeneity in dosing among reported cases, and the lack of controlled trials in KCNQ2-associated epilepsy.

In summary, we performed a large-scale in vitro functional and pharmacological evaluation of KCNQ2 variants in the homozygous and heterozygous states using automated patch clamp recordings. Our findings contribute to understanding the spectrum of KCNQ2 channel dysfunction in monogenic epilepsy and reveal variable retigabine responsiveness among the studied variants. We also observed that dominant-negative missense variants are not exclusive to KCNQ2-associated DEE. The validated functional assay we employed in this study provided data that can be used to help distinguish true pathogenic from nonpathogenic variants and serve as a platform for evaluating drug responses of other KCNQ2 variants.

## Methods

### Variant identification and prioritization for study.

To enable validation and deployment of the automated patch recording pipeline, we prioritized 81 variants from de-identified data provided by a clinical testing laboratory (GeneDX), public databases (Human Gene Mutation Database [HGMD], gnomAD, RIKEE), and de-identified clinical research databases maintained at Baylor College of Medicine and the Children’s Hospital of Philadelphia. Variants were sorted into categories based on availability of phenotypes, recurrence, location in pathogenic hotspots (13, 14, 28), and previously published functional characterization. Our study included variants with well-described phenotypes, including BFNE, neonatal-onset DEE, and later-onset DEE with variable seizure severity, and previous comprehensive evaluations by voltage-clamp electrophysiology. Additional sets of variants with incompletely understood genotype-phenotype relationships were also selected. These variants included several highly recurrent variants not yet described in published functional studies, rare variants for which clinical phenotyping was less extensive, and variants where earlier voltage-clamp study was fragmentary or merited retesting due to atypical or conflicting results. Based on gnomAD, we also selected 23 rare variants (minor allele frequency ranging from 0.000008 to 0.003) and a single *KCNQ2* common variant (N780T; Supplemental Table 1). All variants were annotated based on the National Center for Biotechnology Information (NCBI) reference sequence NM_172104.

### Cell culture.

Chinese hamster ovary cells (American Type Culture Collection, CHO-K1, CRL 9618) were grown in F-12 nutrient mixture medium (GIBCO, Invitrogen) supplemented with 10% FBS (Atlanta Biologicals), penicillin (50 units/mL^−1^), and streptomycin (50 μg/mL^−1^) at 37°C in 5% CO_2_.

A cell line (CHO-Q3) stably expressing human WT KCNQ3 (Q3, GenBank accession number NM_004519) was generated using Flp-In-CHO cells according to the supplier’s protocol (Thermo Fisher Scientific). CHO-Q3 stable cells were maintained under dual selection with Zeocin (100 μg/mL) and hygromycin B (600 μg/mL) in F-12 nutrient mixture medium (GIBCO, Invitrogen) supplemented with 10% FBS (Atlanta Biologicals), penicillin (50 units/mL^−1^), and streptomycin (50 μg/mL^−1^) at 37°C in 5% CO_2_. Q3-expressing clones were screened by electroporating WT KCNQ2 and measuring M-current density by automated patch clamp recording (see below). One clone (L1) exhibiting the highest percentage of cells expressing M-current (defined as XE991-sensitive current) was selected for experiments. The cell line was only used between passage number 4 and 15.

Unless otherwise stated, all tissue culture media were obtained from Life Technologies.

### Plasmids and transfection.

A cDNA encoding the complete open reading frame of KCNQ3 was cloned into pcDNA5/FRT for use in generating the CHO-Q3 stable cell line. Other vectors were designed to enable coexpression of untagged channel subunits with fluorescent proteins as a means for tracking successful cell transfection. A cDNA with the complete coding region of human KCNQ2 (GenBank accession number NM_172108), which is an abundant splice isoform in human brain (56) was engineered in the mammalian expression vector pIRES2-EGFP (BD Biosciences-Clontech). The EGFP-expressing vector was used for transfection of WT or variant KCNQ2 into CHO-Q3 cells (homozygous state). For studies designed to investigate KCNQ2 variants in the heterozygous state, CHO-Q3 cells were cotransfected with the KCNQ2 variant in the EGFP-expressing vector and KCNQ2-WT in a modified vector where EGFP was substituted with the cyan-excitable orange-red fluorescent protein CyOFP1 (57).

Of the KCNQ2 variants, 3 (A193D, R201C, and P335L) yielded too few positively transfected cells for automated patch clamp recording when expressed as homozygous channels in CHO-Q3 cells. We overcame this limitation by transiently cotransfecting CHO-K1 cells with each of these variants along with KCNQ3, which was expressed using a modified pIRES2-vector coexpressing the monomeric red fluorescent protein mScarlet (58). These variants were successfully analyzed using CHO-Q3 cells when expressed as heterozygous channels.

### Site-directed mutagenesis.

KCNQ2 variants were introduced into the WT coding sequence using Q5 High Fidelity DNA polymerase (New England Biolabs). Mutagenic primers for each variant were designed using custom software (Q5 Designer). Mutagenic primer sequences are provided in Supplemental Table 7). Variant KCNQ2 plasmid clones were screened as previously described for the related potassium channel KCNQ1 (58). For each variant plasmid, the complete KCNQ2 coding region, the IRES element, and fluorescent protein cDNA were sequenced in their entirety by Sanger sequencing (Eurofins Genomics) and analyzed using Multiple Sequence Iterative Comparator (MuSIC), a custom multiple sequence alignment tool. Endotoxin-free plasmid DNA was purified from clones with correct sequences (Nucleobond Xtra Maxi EF, Macherey-Nagel) and resuspended in endotoxin-free water.

### Electroporation.

Plasmids encoding WT or variant KCNQ2 channels were transiently transfected by electroporation using the MaxCyte STX system (59). CHO-Q3 or CHO-K1 cells grown to 70%–80% confluence were harvested using 0.25% trypsin. A 500 μL aliquot of cell suspension was then used to determine cell number and viability using an automated cell counter (ViCell, Beckman Coulter). Remaining cells were collected by gentle centrifugation (160*g*, 4 minutes at room temperature), washed with 5 mL electroporation buffer (EBR100, MaxCyte), and resuspended in electroporation buffer at a density of 10^8^ viable cells/mL. Each electroporation was performed using 100 μL of cell suspension.

CHO-Q3 cells were electroporated with 15 μg of WT or variant KCNQ2 cDNA for homozygous channel studies or with 10 μg of each variant and WT KCNQ2 cDNAs for experiments testing the heterozygous state. For the 3 variants tested by coexpression with KCNQ3 in CHO-K1 cells, 15 μg of variant or WT KCNQ2 cDNA and 35 μg of KCNQ3 cDNA were used per electroporation. The DNA-cell suspension mix was transferred to an OC-100 processing assembly (MaxCyte) and electroporated using the preset CHO-PE protocol. Immediately after electroporation, 10 μL of DNase I (Sigma-Aldrich) was added to the DNA-cell suspension and the entire mixture was transferred to a 35 mm tissue culture dish for a 30 minute incubation at 37°C in 5% CO_2_. Following incubation, cells were gently resuspended in culture media, transferred to a T75 tissue culture flask and grown for 48 hours at 37°C in 5% CO_2._ Following incubation, cells were harvested, counted, transfection efficiency determined by flow cytometry (see below), and then frozen in 1 mL aliquots at 1.510^6^ viable cells/mL in liquid N_2_ until used in experiments.

### Flow cytometry.

Transfection efficiency was evaluated by flow cytometry (CytoFLEX, Beckman Coutler) using a 488 nm laser. Forward scatter (FSC), side scatter (SSC), FITC, EGFP, orange fluorescence (PEA, CyOFP) and red fluorescence (PE, mScarlet) were recorded. FSC and SSC were used to gate single viable cells and to eliminate doublets, dead cells, and debris. A total of 10,000 events were recorded for each sample. Nonelectroporated cells were assayed as control for all parameters to set the gates for each experiment, and the percentage of fluorescent cells was determined from the gated population. Compensation matrixes were generated using CHO-Q3 or CHO-K1 cells expressing only single fluorescent markers and applied to the cotransfected cells to account for spectrum overlap. The percentage of cotransfected cells was determined from plots of FITC versus PEA or FITC versus PE fluorescence intensity.

### Cell preparation for automated electrophysiology.

Electroporated cells were thawed the day before the experiments, plated, and incubated for 10 hours at 37°C in 5% CO_2_. The cells were then grown overnight at 28°C in 5% CO_2_ to increase channel expression at the plasma membrane. Prior to experiments, cells were passaged using 5% trypsin in cell culture media. Cell aliquots (500 μL) were used to determine cell number and viability by automated cell counting and transfection efficiency by flow cytometry. Cells were then diluted to 200,000 (single-hole chips) or 600,000 (4-hole chips) cells/mL with external solution (see below) and allowed to recover 60 minutes at 15°C while shaking on a rotating platform at 200 rpm.

### Automated patch clamp recording.

Automated patch clamp recording was performed using the Syncropatch 768 PE platform (Nanion Technologies) as previously described (58, 59). Experiments were performed blinded to variant phenotype or source through to the completion of data analysis. Single-hole, 384-well recording chips with medium resistance (2–4 MΩ) were used for most experiments, and 4-hole, 384-well recording chips with medium resistance were used to investigate TEA dose-response relationships. The external solution contained (in mM): NaCl 140, KCl 4, CaCl_2_ 2, MgCl_2_ 1, HEPES 10, and glucose 5, with the final pH adjusted to 7.4 with NaOH. The internal solution contained (in mM): KF 60, KCl 50, NaCl 10, HEPES 10, EGTA 20, and ATP-Mg 5, with the final pH adjusted to 7.2 with KOH. The calculated intracellular free Mg^2+^ concentration is 0.57 mM.

Pulse generation and data collection were carried out with PatchController384 V.1.3.0 software (Nanion Technologies). Whole-cell currents recorded at room temperature in the whole-cell configuration were filtered at 3 kHz and acquired at 10 kHz. The access resistance and apparent membrane capacitance were estimated using built-in protocols. Access resistance compensation was set to 80%. Stringent criteria were used to include individual recordings for final data analysis. We required seal resistance to be greater or equal to 0.5 GΩ except for recordings performed after application of XE991 where we used a threshold of 0.3 GΩ and recordings done with 4-hole chips in which the criterion for inclusion was greater than 50 MΩ. The criteria for series resistance and capacitance were less than or equal to 20 MΩ and greater or equal to 1 picofarad, respectively. A criterion for voltage-clamp stability was defined by a standard error of less than 10% for baseline current measured at the holding potential for all test pulses. Whole-cell currents were not leak-subtracted. The contribution of background currents was determined by recording before and after addition of the M-current blocker XE991 (25 μM, Abcam; or TOCRIS). The currents recorded in XE991 were digitally subtracted from recordings done in the absence of XE991. To generate maximum XE991 block, 8–12 30 second depolarizing voltage pulses to +10 mV were applied at 0.025 Hz before recording. None of the KCNQ2 variants tested in this study exhibited insensitivity to XE991 under these conditions, and there was no appreciable block of endogenous outward currents in CHO-Q3 cells.

Currents were elicited from a holding potential of –80 mV using 1000 milliseconds of depolarizing pulses (from –80 mV to +40 mV in +10mV steps, 0.05 Hz) followed by a 250 millisecond step to 0 mV to analyze tail currents. Maximum current amplitude was measured 999 milliseconds after the start of each depolarizing voltage pulse whereas tail currents were measured 5 milliseconds after changing the membrane potential to 0 mV. Voltage protocols were repeated in the presence of retigabine (10 μM; Sigma-Aldrich).

### Whole-cell patch clamp recording.

Manual patch recordings were obtained using an Axon 200B amplifier and pClamp 9 software (Molecular Devices) as previously described (50). Electroporated cells were thawed and plated onto glass cover slips, then maintained initially at 37°C and subsequently at 28°C as described for automated patch clamp experiments. Recordings were obtained using the same internal solution used for automated patch clamp and with internal solutions used previously for manual recordings (50). There were no differences in channel behavior between the 2 internal solutions.

### Data analysis.

Data were analyzed and plotted using a combination of DataController384 version 1.8 (Nanion Technologies), Excel (Microsoft Office 2013, Microsoft), SigmaPlot 2000 (Systat Software) and Prism 8 (GraphPad Software). Whole-cell currents were normalized for membrane capacitance and results expressed as mean ± SEM. The voltage-dependence of channel activation was determined only for cells with mean current density values greater than the background current amplitude. Tail currents were normalized to maximal (peak) tail current amplitude and expressed as a function of the preceding voltages. Data were fit to a Boltzmann function I(V) = Imax/(1 + exp[(1/*k*) − (V − V½)]), where Imax is the maximum tail current amplitude at test potential V, V½ is the half-maximal activation potential, and *k* is a slope factor. The time-constant of activation (τ) was determined by fitting currents elicited by voltage steps between –30 mV and +40 mV (50–1000 ms after start of the voltage step) to a single exponential function: *A*(*t*) = *A*_0_ × exp(−*t*/τ) + C, where *A*_0_ is the signal amplitude, *t* is time in milliseconds, τ is the time constant of activation, and C is the signal offset.

Typical experiments compared 5 KCNQ2 variants to the WT channels assayed on the same plate with up to 64 replicate recordings. Properties of each KCNQ2 variant are presented relative to the WT channel assayed in parallel as percent current density measured at +40 mV, difference (Δ) in voltage-dependence of activation V½, and the ratio of activation time-constants (variant/WT). The number of cells used for each experimental condition is given in the figure legends or in Supplemental Tables.

### Statistics.

Statistical analysis was performed with 1-way ANOVA (2 or more variants) or 2-tailed *t* test, and *P* ≤ 0.01 was considered significant.

### Study approval.

All human data were collected after informed consent as part of routine clinical care at GeneDx or under IRB research protocols approved by the Institutional Review Board for Human Subject Research for Baylor College of Medicine and Affiliated Hospitals or Children’s Hospital of Philadelphia Committees for the Protection of Human Subjects and were de-identified prior to consideration for this study.

### Data availability.

The authors confirm that the data supporting the findings of this study are available within the article and its Supplementary material.

## Author contributions

CGV, ECC, IH, and ALG conceived and designed the study. EF, KLH, DM, ASL, FZ, IH, N Joshi and ECC identified and prioritized variants for study. CGV, RRD, ZJ, SA, N Jairam, and NG performed experiments, collected and analyzed data. CGV and ALG assembled figures and tables. CGV, ECC, and ALG wrote and revised the manuscript. ALG secured funding for the study. All authors approved the manuscript.

## Supplementary Material

Supplemental data

Supplemental table 2

Supplemental table 3

Supplemental table 4

Supplemental table 5

Supplemental table 6

## Version 1 02/01/2022

In-Press Preview

## Version 2 03/08/2022

Electronic publication
